# Revision of the genus *Ptomaphagus* Hellwig (Coleoptera, Leiodidae, Cholevinae) from Taiwan Island

**DOI:** 10.3897/zookeys.609.9327

**Published:** 2016-08-08

**Authors:** Cheng-Bin Wang, Masaaki Nishikawa, Michel Perreau, Jan Růžička, Yasuhiko Hayashi

**Affiliations:** 1 Department of Ecology, Faculty of Environmental Sciences, Czech University of Life Sciences Prague, Kamýcká 129, CZ-165 21 Praha 6, Czech Republic; 2 Kashiwagaya 1112–16, Ebina, 243–0402 Japan; 3 IUT Paris Diderot, Université Paris Diderot case 7139 Sorbonne Paris Cité, 5, rue Thomas Mann, 75205 Paris cedex 13; 4 Suimeidai 3-1-73, Kawanishi, 666-0116 Japan

**Keywords:** Leiodidae, Cholevinae, Ptomaphagus, taxonomy, new species, new synonym, Taiwan

## Abstract

Ptomaphagus (s. str.) chenggongi **sp. n.** and Ptomaphagus (s. str.) tingtingae **sp. n.** (Coleoptera, Leiodidae, Cholevinae, Ptomaphagini) are described from Taiwan Island. In addition, a new subjective synonym is proposed, Ptomaphagus (s. str.) yasutoshii Nishikawa, 1993 = Ptomaphagus (s. str.) smetanai Perreau, 1996, **syn. n.** Relevant morphological characters of the examined *Ptomaphagus* species are illustrated with colour plates, and their known distributions are mapped.

## Introduction

*Ptomaphagus* Hellwig, 1795 is the most speciose genus (including 136 known species worldwide) in the tribe Ptomaphagini (Coleoptera, Leiodidae, Cholevinae). However, the nominotypical subgenus, which is limited to the Palaearctic and north Oriental regions, has only 28 species (Perreau 2000, Nishikawa 2011, Wang et al. in press).

On the island of Taiwan, only three species in the subgenus *Ptomaphagus* s. str. had been recorded before this study, namely Ptomaphagus (s. str.) kuntzeni Sokolowski, 1957, Ptomaphagus (s. str.) yasutoshii Nishikawa, 1993 and Ptomaphagus (s. str.) smetanai Perreau, 1996.

In this paper, which is a continuation of our revision of *Ptomaphagus* from East Asia, two new species with very similar aedeagi from Taiwan are described and illustrated: Ptomaphagus (s. str.) chenggongi sp. n. and Ptomaphagus (s. str.) tingtingae sp. n. In addition, after examination of the holotypes, a new subjective synonym is proposed: Ptomaphagus (s. str.) yasutoshii Nishikawa, 1993 = Ptomaphagus (s. str.) smetanai Perreau, 1996, syn. n. The geographic variation on apicoventral piece of aedeagal median lobe between the populations of Ptomaphagus (s. str.) kuntzeni from Japan and Taiwan is mentioned. Relevant morphological characters of the examined *Ptomaphagus* species are illustrated with colour plates, and their known distributions are mapped.

## Material and methods

Specimens were relaxed and softened in a hot saturated solution of potassium hydroxide for 4 minutes (for mounted dry specimens) or 8 minutes (for alcohol-preserved specimens), and then transferred to distilled water to rinse the residual potassium hydroxide off and stop any further bleaching. The softened specimens were moved into glycerine and dissected there to observe morphological details. After examination, the body parts were mounted on a glass slip with Euparal Mounting Medium for future studies. Habitus photographs were taken using a Canon macro photo lens MP-E 65mm on a Canon 550D. Observations, photographs and measurements of morphological details were performed using an Axio Zoom.V16 motorized stereo zoom microscope with an AxioCam MRc 5 in Beijing, or an Olympus BX53 microscope with an Olympus DP73 in Prague. The final deep focus images were created with Helicon Focus 5.3 stacking software in Beijing or Zerene Stacker 1.04 in Prague. The program Adobe Photoshop CS6 was used for post processing. Exact label data are cited for all specimens examined. Authors’ remarks and addenda are placed in square brackets, separate label lines are indicated by a slash (/) and separate labels by a double slash (//). Measurements are mean values based on 5 specimens.

The material examined for this study is deposited in the following collections and museums:

CCBW
 Collection of Cheng-Bin Wang, Chengdu, Sichuan, China 


CJRZ
 Collection of Jan Růžička, Prague, Czech Republic 


CMNE
 Collection of Masaaki Nishikawa, Ebina, Japan 


CMPR
 Collection of Michel Perreau, Paris, France 


CYHK
 Collection of Yasuhiko Hayashi, Kawanishi, Japan 


MHNG
Muséum d’Histoire Naturelle, Genève, Switzerland (G. Cuccodoro) 


NSMT
National Museum of Nature and Science, Tsukuba, Japan (S. Nomura) 


NTUC
National Taiwan University, Taipei, Taiwan, China 


SMNS
Staatliches Museum für Naturkunde, Stuttgart, Germany (W. Schawaller) 


ZMHB
Museum für Naturkunde – Leibniz-Institut für Evolutions- und Biodiversitätsforschung an der Humboldt-Universität zu Berlin, Berlin, Germany (J. Frisch) 


The following abbreviations are used for the measurements in millimetres (mm):

AL
 (antennal length): length from the antennal base to apex.

BTW
 (basitarsal width): maximum width of 1st protarsomere.

EBL
 (extended body length): summation of HL, PL, ELL and length of exposed scutellum, preventing the error introduced by exposed or retracted head.

ELL
 (elytral length): length from the tail end of scutellum to the elytral apex.

ELW
 (elytral width): maximum width of two elytra combined together.

EW
 (eye width): width of a single compound eye in dorsal view.

HL
 (head length): axial length from the anterior apex of clypeus through the posterior margin of occipital carina.

HW
 (head width): maximum width of head (usually including eyes).

PL
 (pronotal length): axial length of the pronotum.

PW
 (pronotal width): maximum width of pronotum.

TW
 (tibial width): maximum width of protibia (excluding spines along outer margin etc.).

## Results

### Genus Ptomaphagus

Taxon classificationAnimaliaColeopteraLeiodidae

Hellwig, 1795

#### Distribution.

Holarctic, north Oriental, north Neotropical.

### Subgenus Ptomaphagus s. str.

Taxon classificationAnimaliaColeopteraLeiodidae

#### Distribution.

Palaearctic, north Oriental.

#### Key to species of *Ptomaphagus* Hellwig from Taiwan Island

**Table:** 

1	Metathoracic wings absent; aedeagus large and strongly asymmetrical, median lobe turning to right at apex (Figs 6A, B); spermatheca spiral-shaped, distal part discoid (Fig. 7A–C)	**Ptomaphagus (s. str.) yasutoshii Nishikawa**
–	Metathoracic wings fully developed; aedeagus small and almost symmetrical, median lobe not turning to right at apex; spermatheca J-shaped, distal part simply curved	**2**
2	Elytral apices widely rounded; aedeagus stout and wide; spermatheca not coiled in proximal part	**Ptomaphagus (s. str.) kuntzeni Sokolowski**
–	Elytral apices narrowly rounded (Figs 8G, H; 11G, H); aedeagus long and slender (Fig. 9A–D); spermatheca coiled in proximal part (Fig. 10B, C)	**3**
3	Antennomere XI with length/width = 1.9 (Fig. 8A); right apicoventral piece of aedeagal median lobe broad (Fig. 9H); spermatheca extended leftwards in proximal part (Fig. 10B)	**Ptomaphagus (s. str.) chenggongi sp. n.**
–	Antennomere XI with length/width = 1.3 (Fig. 11A); right apicoventral piece of aedeagal median lobe rather small (Fig. 9I); spermatheca not extended leftwards in proximal part (Fig. 10C)	**Ptomaphagus (s. str.) tingtingae sp. n.**

**Figure 1.**
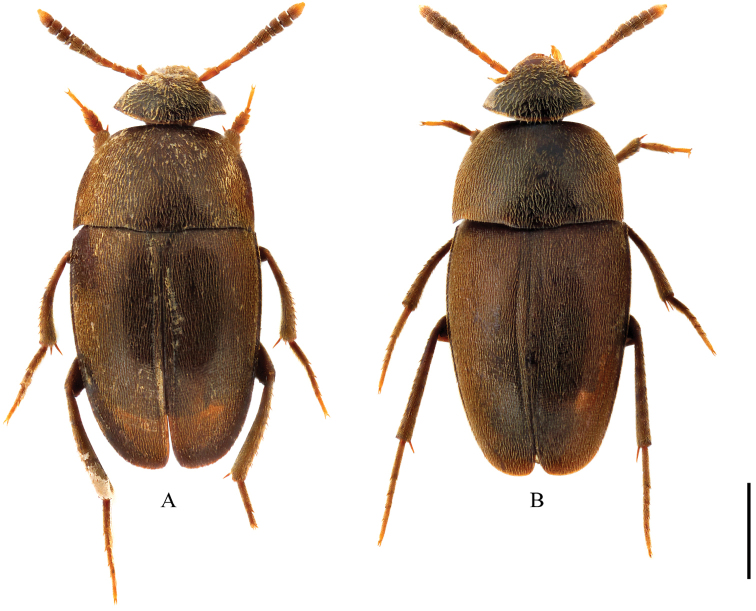
Habitus of Ptomaphagus (s. str.) kuntzeni Sokolowski, 1957 from Taiwan (dorsal view). **A** ♂ (Fenchihu) **B** ♀ (Meifeng). Scale bar 1 mm.

**Figure 2.**
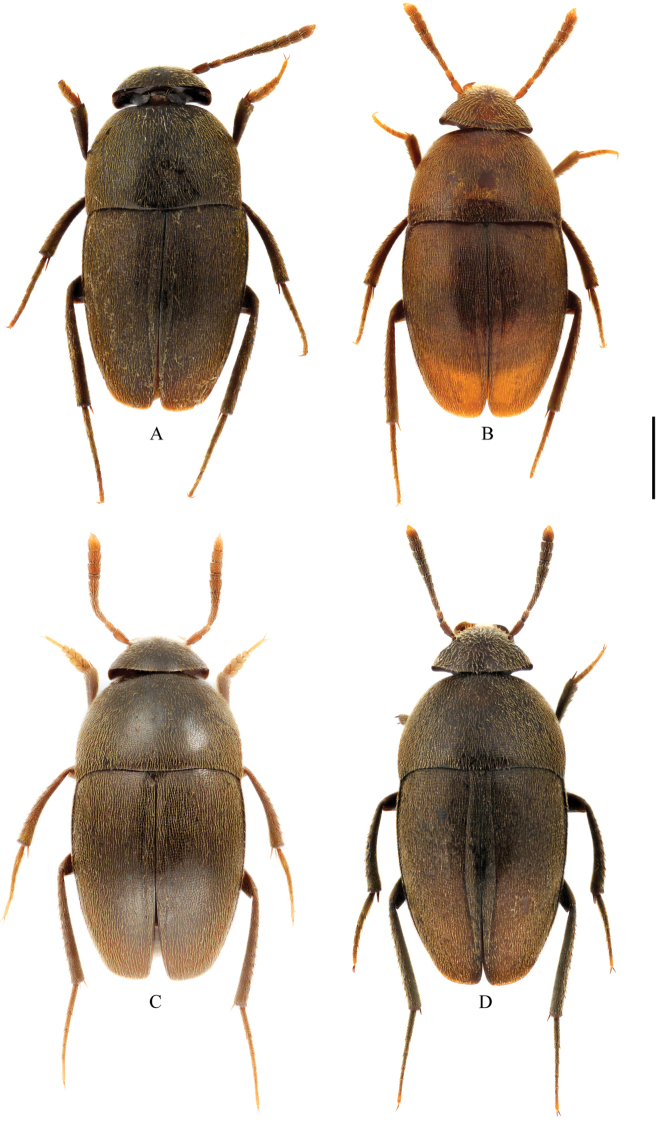
Habitus of Ptomaphagus (s. str.) yasutoshii Nishikawa, 1993 (dorsal view). **A** ♂ (holotype) **B** ♀ (allotype) **C** ♂ (holotype of Ptomaphagus (s. str.) smetanai Perreau, 1996, syn. n.) **D** ♀ (Yushan). Scale bar 1 mm.

**Figure 3.**
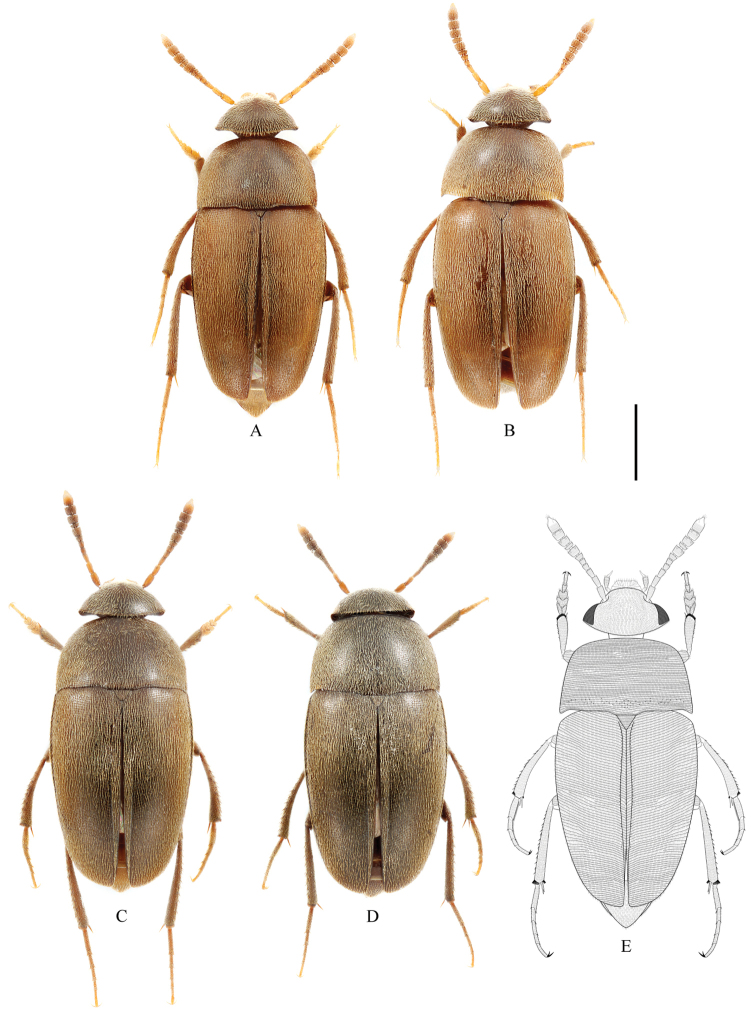
Habitus of *Ptomaphagus* (s. str.) spp. (dorsal view). **A, B** Ptomaphagus (s. str.) chenggongi sp. n. **C–E** Ptomaphagus (s. str.) tingtingae sp. n. **A, C** ♂ (holotypes) **B, D** ♀ (paratypes) **E** ♂ (line-art drawing). Scale bar 1 mm.

**Figure 4.**
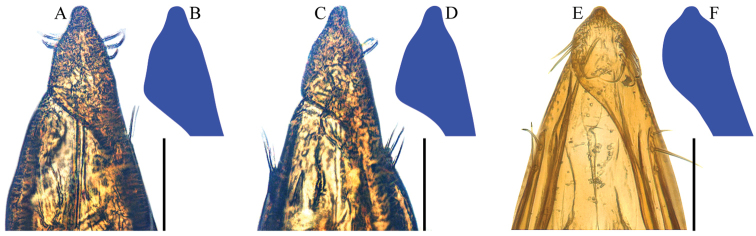
Aedeagal apices of Ptomaphagus (s. str.) kuntzeni Sokolowski, 1957 (ventral view). **A–D** Fenchihu, Taiwan Island **E, F** Amami-Ôshima Island, Japan **B, D, F** right apicoventral piece of median lobe. Scale bars 0.1 mm.

**Figure 5.**
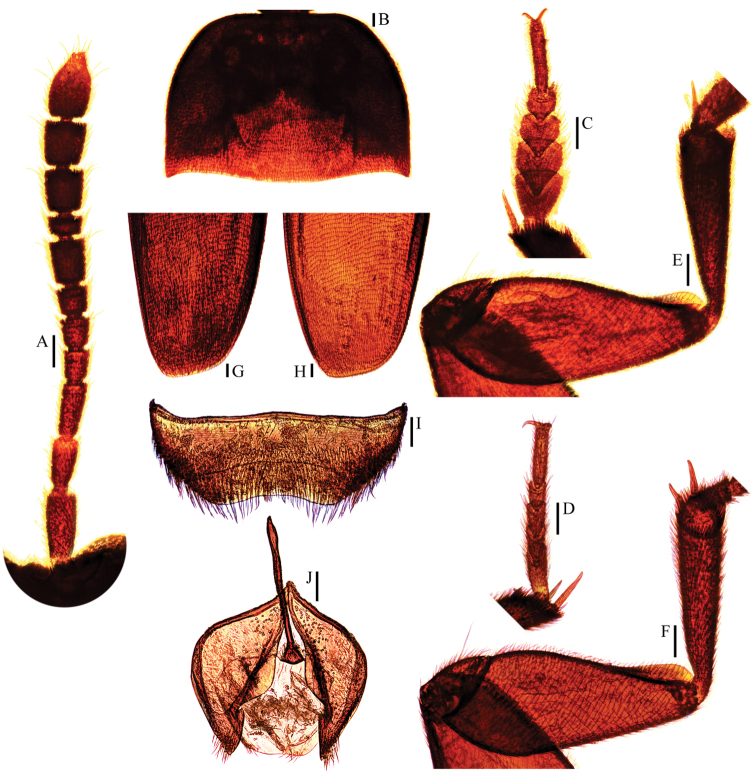
Ptomaphagus (s. str.) yasutoshii Nishikawa, 1993 (♂ holotype; ♀ allotype). **A** antenna ♂ (dorsal view) **B** pronotum ♂ (dorsal view) **C** protarsus ♂ (dorsal view) **D** protarsus ♀ (dorsal view) **E** protibia and profemur ♂ (ventral view) **F** protibia and profemur ♀ (ventral view) **G** elytral apex ♂ (dorsoapical view) **H** elytral apex ♀ (dorsoapical view) **I** ventrite VIII ♂ (ventral view) **J** genital segment ♂ (ventral view). Scale bars 0.1 mm.

**Figure 6.**
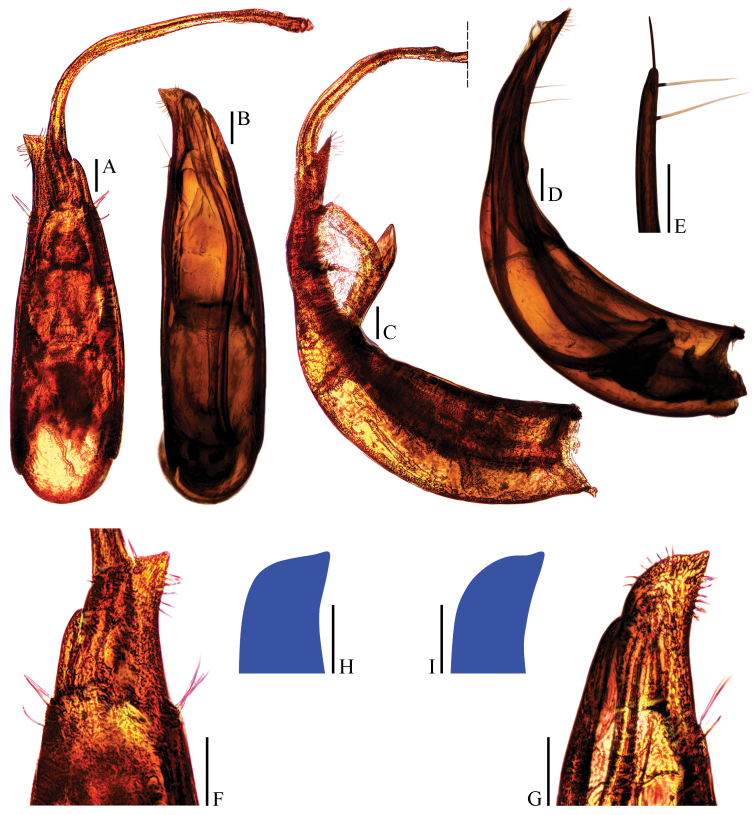
Aedeagi of Ptomaphagus (s. str.) yasutoshii Nishikawa, 1993. **A, C, F, H** holotype **B, D, E, G, I** holotype of Ptomaphagus (s. str.) smetanai Perreau, 1996, syn. n. **A, B** aedeagi (dorsal view) **C, D** aedeagi (lateral view) **E** paramere apex (lateral view) **F, G** aedeagal apices (ventral view) **H, I** right apicoventral piece of median lobe (ventral view). Scale bars 0.1 mm.

**Figure 7.**
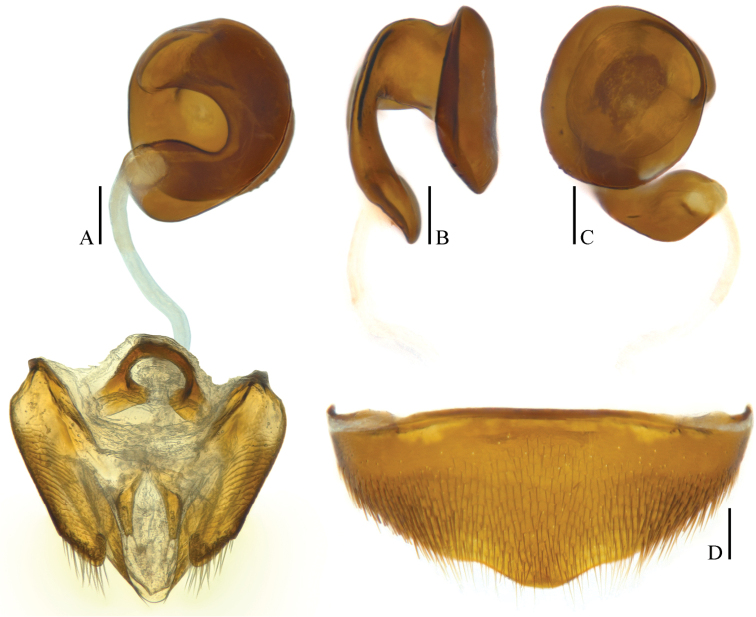
Ptomaphagus (s. str.) yasutoshii Nishikawa, 1993 (♀ Yushan). **A** spermatheca and genital segment (ventral view) **B** spermatheca (lateral view) **C** spermatheca (dorsal view) **D** ventrite VIII ♀ (ventral view). Scale bars 0.1 mm.

**Figure 8.**
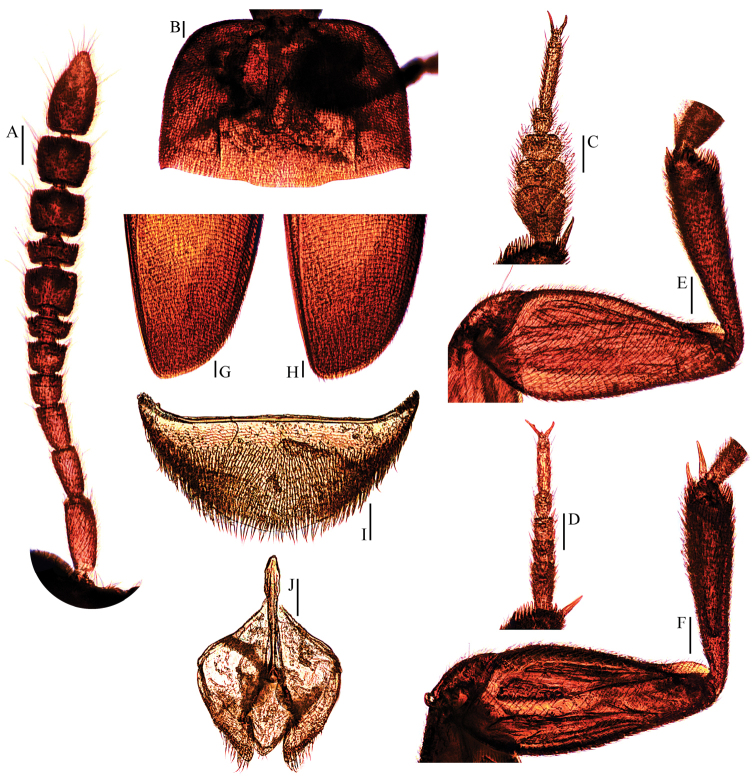
Ptomaphagus (s. str.) chenggongi sp. n. (paratypes). **A** antenna ♂ (dorsal view) **B** pronotum ♂ (dorsal view) **C** protarsus ♂ (dorsal view) **D** protarsus ♀ (dorsal view) **E** protibia and profemur ♂ (ventral view) **F** protibia and profemur ♀ (ventral view) **G** elytral apex ♂ (dorsoapical view) **H** elytral apex ♀ (dorsoapical view) **I** ventrite VIII ♂ (ventral view) **J** genital segment ♂ (ventral view). Scale bars 0.1 mm.

**Figure 9.**
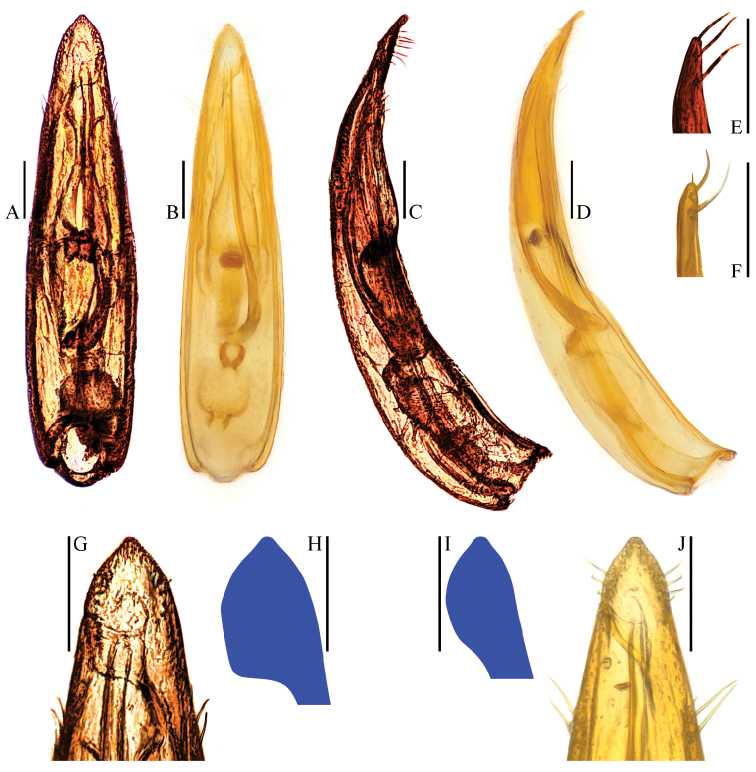
Aedeagi of *Ptomaphagus* (s. str.) spp. **A, C, E, G, H** Ptomaphagus (s. str.) chenggongi sp. n. (paratype) **B, D, F, I, J** Ptomaphagus (s. str.) tingtingae sp. n. (paratype) **A, B** aedeagi (dorsal view) **C, D** aedeagi (lateral view) **E, F** paramere apices (lateral view) **G, J** aedeagal apices (ventral view) **H, I** right apicoventral piece of median lobe (ventral view). Scale bars 0.1 mm.

**Figure 10.**
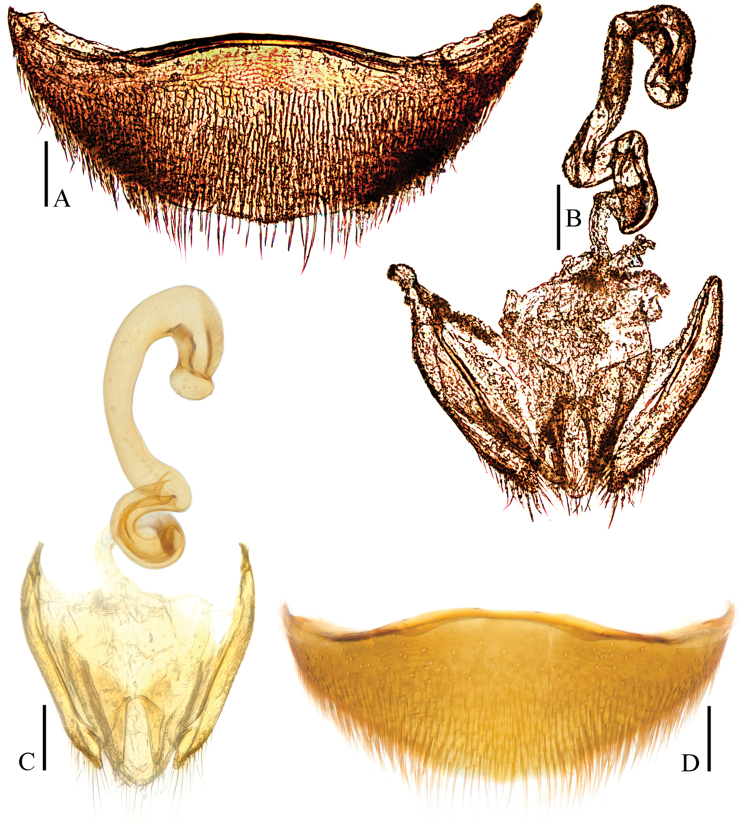
**A, B** Ptomaphagus (s. str.) chenggongi sp. n. (paratype) **C, D** Ptomaphagus (s. str.) tingtingae sp. n. (paratype) **A, D** ventrites VIII (ventral view) **B, C** spermathecae and genital segments (ventral view). Scale bars 0.1 mm.

**Figure 11.**
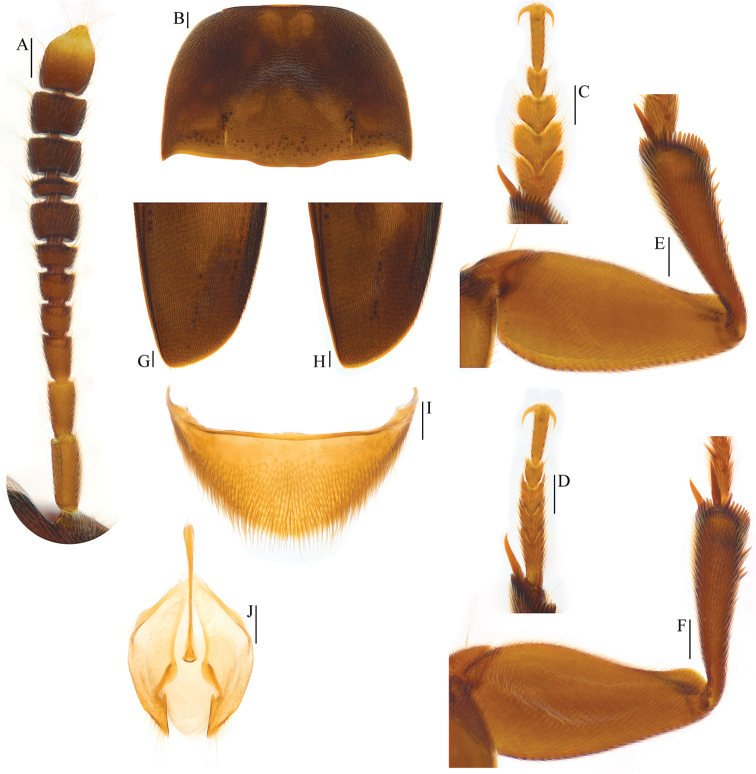
Ptomaphagus (s. str.) tingtingae sp. n. (paratypes). **A** antenna ♂ (dorsal view) **B** pronotum ♂ (dorsal view) **C** protarsus ♂ (dorsal view) **D** protarsus ♀ (dorsal view) **E** protibia and profemur ♂ (dorsal view) **F** protibia and profemur ♀ (dorsal view) **G** elytral apex ♂ (dorsoapical view) **H** elytral apex ♀ (dorsoapical view) **I** ventrite VIII ♂ (ventral view) **J** genital segment ♂ (ventral view). Scale bars 0.1 mm.

**Figure 12.**
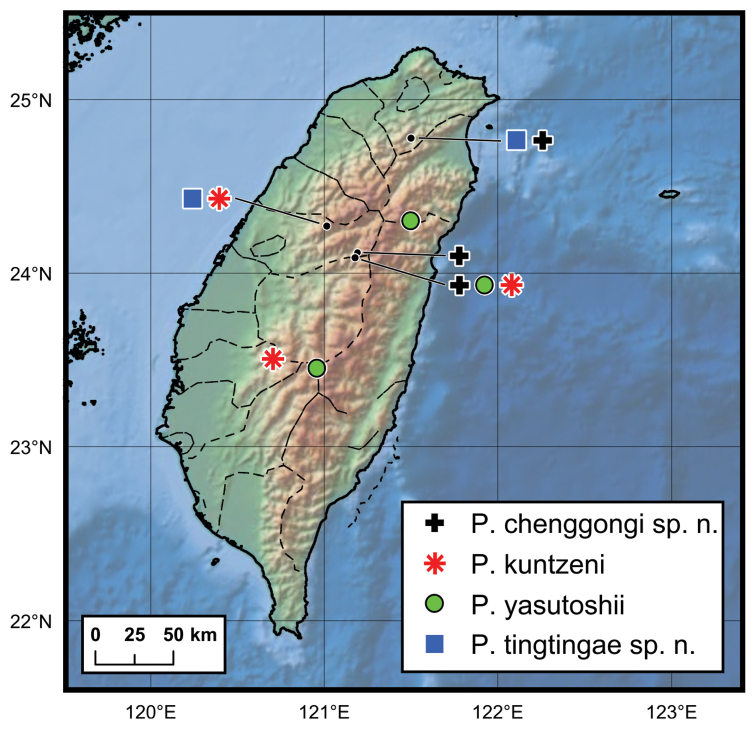
Distribution map of *Ptomaphagus* species from Taiwan Island.

### Ptomaphagus (s. str.) kuntzeni

Taxon classificationAnimaliaColeopteraLeiodidae

Sokolowski, 1957

Figs 1 ; 4

Ptomaphagus (s. str.) kuntzeni Sokolowski 1957: 140 (Ptomaphagus; type locality: [JAPAN] Hagi (? Landschaft Jamagutshi, Honshiu); ZMHB); Szymczakowski 1964: 63 (Ptomaphagus; female description; taxonomic remarks); Nishikawa 1983: 1 (Ptomaphagus (Ptomaphagus); in check-list); Harusawa and Yamamoto 2000: 242 (Ptomaphagus; distribution); Hayashi and Nishikawa 2010: 190 (Ptomaphagus; distribution); Perreau 2000: 363 (Ptomaphagus (s. str.); in catalogue); Perreau 2004: 178 (Ptomaphagus (Ptomaphagus); in catalogue); Nishikawa 2011: 100 (Ptomaphagus (Ptomaphagus); distribution; notes); Nishikawa et al. 2012: 274 (Ptomaphagus (Ptomaphagus); distribution); Perreau 2015: 249 (Ptomaphagus (Ptomaphagus); in catalogue); Wang et al. in press (Ptomaphagus (s. str.); redescription; distribution; remarks).
Ptomaphagus (s. str.) amamianus Nakane 1963: 42 (Ptomaphagus; type locality: [JAPAN] Naze, Amami-Oshima); Hayashi 1969: 2 (Ptomaphagus; characteristic figures; distribution); Nishikawa 1983: 1 (Ptomaphagus (Ptomaphagus); in check-list); Perreau 1996: 284 (Ptomaphagus; distribution); Perreau 2000: 362 (Ptomaphagus (s. str.); in catalogue); Perreau 2004: 178 (Ptomaphagus (Ptomaphagus); in catalogue); Hayashi and Nishikawa 2010: 190 (Ptomaphagus; distribution); Perreau 2015: 249 (Ptomaphagus (Ptomaphagus); in catalogue); Wang et al. in press (Ptomaphagus (s. str.); synonymy with Ptomaphagus kuntzeni).

#### Material examined.

3♂♂, Taiwan, 25.V.1977 / Fenchihu [奮起湖, ca. 23°30'N, 120°42'E], 1400 m / Klapperich leg. (1♂ in CJRZ, 1♂ in CMPR and 1♂ in MHNG); 1♀, same data as previous except: 14.V.1977 (CMPR); 1♀, TAIWAN, Nantou / Hsien, Meifeng [梅峰, ca. 24°05'N, 121°10'E] / 2130 m 10–17.VII.[19]93 / yellow pan traps / A. Smetana [leg.] (T147) (CJRZ); 1♀, TAIWAN Taichung / Hsien, Anmashan [鞍馬山, ca. 24°16'N, 121°00'E] / 2230 m 30.IV.–4.V.[19]90 / A. Smetana [leg.] (T32) // PTOMAPHAGUS / sp. (SMNS).

#### Remarks.

Perreau (1996) recorded the species from Taiwan Island under the name Ptomaphagus (s. str.) amamianus. We re-examined the specimens concerned and found that they have a partly different aedeagus from that of Japanese specimens (Fig. 4A, C, E): in specimens from Taiwan, the right apicoventral piece of median lobe is slender and subtriangular (Fig. 4B, D); while in specimens from Japan, it is shorter and subround (Fig. 4F). However, we consider that it is an intraspecific geographic variation because they have the basically identical shape of aedeagi and spermathecae, and no distinct differences in their external morphology.

#### Distribution.

China (Taiwan) (Fig. 12), Japan, ?Myanmar.

### Ptomaphagus (s. str.) yasutoshii

Taxon classificationAnimaliaColeopteraLeiodidae

Nishikawa, 1993

Figs 2 ; 5 , 6 , 7

Ptomaphagus (s. str.) yasutoshii Nishikawa 1993: 123 (Ptomaphagus (s. str.); type locality: Taiwan, Nantou Hsien, near Tsuifeng, 2200 m; NSMT); Perreau 2000: 367 (Ptomaphagus (s. str.); in catalogue); Perreau 2004: 178 (Ptomaphagus (Ptomaphagus); in catalogue); Perreau 2015: 250 (Ptomaphagus (Ptomaphagus); in catalogue).
Ptomaphagus (s. str.) smetanai Perreau 1996: 285 (Ptomaphagus (s. str.); type locality: Taiwan, Hualien Hsien, Taroko, N. P. Duodyatunshan, 2660 m; MHNG); Perreau 2000: 364 (Ptomaphagus (s. str.); in catalogue); Perreau 2004: 178 (Ptomaphagus (Ptomaphagus); in catalogue); Perreau 2015: 249 (Ptomaphagus (Ptomaphagus); in catalogue). **Syn. n.**

#### Material examined.

**Type material. Holotype** of ***Ptomaphagus yasutoshii***: ♂, (Near TSIFENG [翠峰, ca. 24°06'N, 121°11'E]) / Nantou - Hsien, / TAIWAN (2200 m) / Aug. 25th, 1974 / Coll. Y. Shibata // HOLOTYPE / Ptomaphagus (s. str.) / yasutoshii / M. Nishikawa, 1993 (NSMT). **Allotype of *Ptomaphagus yasutoshii***: ♀, (Near TSIFENG [翠峰, ca. 24°06'N, 121°11'E]) / Nantou - Hsien, / TAIWAN (2200 m) / July 27th, 1974 / Coll. Y. Shibata // ALLOTYPE / Ptomaphagus (Ptomaphagus) / yasutoshii M. Nishikawa, / 1993 / Design. M. Nishikawa, 1993 / # MNIC 124929Ch2S ♀ // ♀ (CMNE). **Holotype of *Ptomaphagus smetanai***: ♂, TAIWAN Hualien / Hsien, Taroko, N. P / [Mt.] Duodyatunshan [多加屯山, ca. 24°18'N, 121°30'E] / 2650 m, 8.–13.V.[19]90 / A. Smetana [leg.] (T57) // HOLOTYPE / PTOMAPHAGUS SMETANAI / M. Perreau det. 1994 // MHNG / ENTO / 00003352 (MHNG).

#### Additional material.

1♀, Mt. YUSHAN [玉山, ca. 23°28'N, 120°57'E] / TAIWAN / 20.V.1981 / N. ITO [leg.] // Ptomaphagus / (s. str.) yasutoshii / MS / Exs. M. NISHIKAWA, 1992 (CYHK).

#### Redescription.

*Male*. EBL: 4.3 mm in holotype of *Ptomaphagus yasutoshii* and 4.9 mm in holotype of *Ptomaphagus smetanai*). Length of different body parts: HL : AL : PL : ELL = 0.78 : 1.51 : 1.08 : 2.25 mm; width: HW : EW : PW : ELW = 1.22 : 0.10 : 1.79 : 1.90 mm. Proportion of antennomeres from base to tip in μm (length × width): 202 × 85, 139 × 81, 170 × 82, 95 × 83, 91 × 98, 87 × 114, 125 × 138, 57 × 122, 117 × 138, 123 × 134, 205 × 119.

*Habitus* (Fig. 2A, C) elongated oval, regularly convex and sublustrous. Well pigmented: mostly dark brown; mouthparts, antennae (apical half of ultimate antennomere yellowish) and tarsi reddish brown. Dorsum continuously clothed with fine, recumbent, yellowish pubescence. Insertions of pubescence on dorsal surfaces of head, pronotum, elytra and femora aligned along transverse striolations; interspace between two striolations glabrous.

*Head* transverse, HW/HL = 1.6. Clypeofrontal suture absent. Clypeus with anterior margin almost straight. Compound eyes small, EW/HW = 0.1. Antennae (Fig. 5A) slender, AL/HW = 1.2; antennomere III much longer than II; VI with length/width = 0.8; XI pear-shaped.

*Pronotum* (Fig. 5B) much transverse, widest just before hind angles, PW/PL = 1.7. Sides regularly rounded, gradually narrowing from posterior to anterior, and slightly constricted before hind angles, which projected backwards and acute. Posterior margin widely protruding in the middle part, distinctly emarginate near hind angles.

*Elytra* oval and quite wide, widest at about basal 1/5, ELL/EW = 1.2. Sides weakly arched, gradually narrowing from widest part to apices, which obliquely truncated (Fig. 5G). Sutural striae present. Metathoracic wings absent.

*Prolegs* robust, with basal three protarsomeres (Fig. 5C) strongly expanded: TW/BTW = 1.1. Protibiae (Fig. 5E) distinctly expanded towards apex. Profemora rather broad. Mesotibiae arcuate, mesotarsi simply linear. Metatibiae slender and straight.

*Abdominal ventrite VIII* (Fig. 5I) widely and deeply emarginate at posterior edge. Genital segment (Fig. 5J) with spiculum gastrale protruding about 1/2 of its length beyond anterior edge of epipleurite IX.

*Aedeagus* (Fig. 6A, B) large, slender and strongly asymmetrical, with median lobe gradually narrowing towards lanceolate apical part which turning to right in dorsal view; opening of genital orifice situated on dorsal surface, deeply cut inwards on preapical left margin of median lobe. Ventral surface of the apical part of the median lobe (Figs 6F, G) inserted with a row of 7 ventrally-oriented setae on both sides. Parameres narrow, reaching about apical 1/5 of median lobe, each with 1 apical and 2 preapical setae, the apical one much shorter (Fig. 6E). In lateral view (Fig. 6C, D), median lobe distinctly bent ventrad and strongly tapering towards narrowly acuminate apex. Endophallus with stylus quite slender, a cheliform complex just below the base of stylus, and a circular complex in basal region.

*Female*. Similar to male in general appearance (Fig. 2B, D), including elytral apices (Fig. 5H), but distinguished by the following characteristics: protarsi (Fig. 5D) simply linear; protibiae (Fig. 5F) much narrower; abdominal ventrite VIII (Fig. 7D) strongly and widely protruded at median of posterior edge; genital segment as shown in Fig. 7A; spermatheca (Fig. 7A–C) spiral-shaped, discoidal in distal part .

#### Remarks.

This species is exceptional in the genus *Ptomaphagus* in the following characters: metathoracic wings absent; aedeagus strongly asymmetrical, median lobe turning rightwards in apical part; spermatheca spiral-shaped, discoidal in distal part.

In addition, the holotypes of *Ptomaphagus yasutoshii* and *Ptomaphagus smetanai* have almost identical aedeagal shape (Fig. 6A–D) and no distinct differences in the external morphology, except some variations exist in the shape of aedeagal apex: the right apicoventral piece of median lobe of *Ptomaphagus yasutoshii* (Fig. 6H) is somewhat wider than that of *Ptomaphagus smetanai* (Fig. 6I). However, such differences, which fall within intraspecific variability, does not prevent us from synonymizing the two species.

#### Distribution.

China (Taiwan) (Fig. 12).

### Ptomaphagus (s. str.) chenggongi sp. n.

Taxon classificationAnimaliaColeopteraLeiodidae

http://zoobank.org/D6994368-3822-4571-8B04-7C3AAF0BC100

Figs 3 ; 8 ; 9A, C, E, G, H ; 10

#### Type locality.

Central Taiwan, Nantou Hsien, Tsuifeng [翠峰, ca. 24°06'N, 121°11'E], 2,300 m.

#### Type material.

**Holotype**: ♂, [Taiwan] Tsuifeng [翠峰, ca. 24°06'N, 121°11'E], 2,300 m / FIT: in shady Forest / Nantou Hsien // Central Taiwan / 7~14-VIII-2003 / Wataru Suzuki leg. (NSMT). **Paratypes**: 22♂♂24♀♀, same data as holotype (22♂♂22♀♀ in CMNE, 2♀♀ in NSMT); 2♂♂, same data as holotype except: FIT: Forest edge (CMNE); 10♂♂10♀♀, same data as holotype except: 2,200 m / FIT: shady natural forest (CMNE); 2♂♂1♀, same data as holotype except: 2,200 m (CMNE); 1♀, TAIWAN, Nantou / Hsien, Meifeng [梅峰, ca. 24°05'N, 121°10'E] / 2130 m 10–17.VII.[19]93 / yellow pan traps / A. Smetana [leg.] (T147) (CJRZ).

#### Description.

*Male*. EBL: 4.1–4.2 mm (4.2 mm in holotype). Length of different body parts: HL : AL : PL : ELL = 0.67 : 1.26 : 1.00 : 2.32 mm; width: HW : EW : PW : ELW = 1.04 : 0.09 : 1.49 : 1.64 mm. Proportion of antennomeres from base to tip in μm (length × width): 179 × 77, 123 × 68, 105 × 74, 75 × 82, 75 × 99, 53 × 113, 104 × 135, 53 × 132, 102 × 138, 117 × 137, 217 × 116.

*Habitus* (Fig. 3A) elongated oval, regularly convex and sublustrous. Well pigmented: mostly brown; mouthparts, basal four or five antennomeres and apical half of ultimate antennomere, protarsi, and apex of meso- and metatarsi yellowish. Dorsum continuously clothed with fine, recumbent, yellowish pubescence. Insertions of pubescence on dorsal surfaces of pronotum, elytra and femora aligned along transverse striolations; interspace between two striolations glabrous.

*Head transverse*, HW/HL = 1.5. Clypeofrontal suture absent. Clypeus with anterior margin slightly rounded. Compound eyes well developed, EW/HW = 0.1. Antennae (Fig. 8A) slender, AL/HW = 1.3; antennomere III a little shorter than II; VI with length/width = 0.5; XI elongated pear-shaped.

*Pronotum* (Fig. 8B) transverse, widest just before hind angles, PW/PL = 1.5. Sides gently arched, narrowing from posterior to anterior, and slightly constricted before hind angles, which projected backwards and subacute. Posterior margin widely protruding in the middle part, distinctly emarginate near hind angles.

*Elytra* oval, widest at about basal 2/7, ELL/EW = 1.4. Sides weakly arched, gradually narrowing from widest part to apices, which narrowly rounded (Fig. 8G). Sutural striae present. Metathoracic wings fully developed.

*Prolegs* robust, with basal three protarsomeres (Fig. 8C) strongly expanded: TW/BTW = 1.0. Protibiae (Fig. 8E) expanded towards apex. Profemora rather broad. Mesotibiae arcuate, mesotarsi simply linear. Metatibiae slender and straight.

*Abdominal ventrite VIII* (Fig. 8I) round at posterior edge, though an inconspicuous median notch at the median. Genital segment (Fig. 8J) with spiculum gastrale protruding about 3/8 of its length beyond anterior edge of epipleurite IX.

*Aedeagus* (Fig. 9A) long and slender, with median lobe gradually narrowing towards lanceolate apical part and terminated by round knob in dorsal view; opening of genital orifice situated on dorsal surface, deeply cut inwards on preapical left margin of median lobe. Ventral surface of the apex of the median lobe (Fig. 9G) inserted with a row of 6 ventrally oriented setae (the bottom one is very short) on the left side and a row of 4 ventrally oriented setae on the right side. Parameres narrow, reaching about apical 1/5 of median lobe, each with 1 apical and 2 preapical setae, the apical one slightly shorter (Fig. 9E). In lateral view (Fig. 9C), median lobe regularly bent ventrad, gradually tapering apically. Endophallus with stylus quite slender, a cheliform complex just below the base of stylus, and a circular complex in basal region.

*Female*. Similar to male in general appearance (Fig. 3B), including elytral apices (Fig. 8H), but distinguished by the following characteristics: protarsi (Fig. 8D) simply linear; protibiae (Fig. 8F) only slightly narrower; abdominal ventrite VIII (Fig. 9A) almost regularly rounded at posterior edge; genital segment as shown in Fig. 9B: spermatheca (Fig. 9B) curved in distal part, coiled and extended leftwards in proximal part.

#### Diagnosis.

Ptomaphagus (s. str.) chenggongi sp. n. has very similar aedeagus to Ptomaphagus (s. str.) tingtingae sp. n., but can be distinguished from the latter by the following characters: in Ptomaphagus (s. str.) chenggongi sp. n., antennomere XI with length/width = 1.9, hind angles of pronotum subacute, spiculum gastrale of genital segment with ordinary width, right apicoventral piece of median lobe broad (Fig. 9H), apical seta of parameres slightly shorter than preapical setae, and spermatheca coiled and extended leftwards in proximal part; while in Ptomaphagus (s. str.) tingtingae sp. n., antennomere XI with length/width = 1.3, hind angles of pronotum acute, spiculum gastrale of genital segment very narrow, right apicoventral piece of median lobe rather small (Fig. 9I), apical seta of parameres very shorter than preapical setae, and spermatheca coiled but not extended leftwards in proximal part.

#### Etymology.

The specific epithet is dedicated to Cheng-Gong Zheng (1624–1662), a military leader at the end of the Chinese Ming Dynasty, for his feats in 1662 when he defeated the forces of the Dutch East India Company and claimed Taiwan, bringing it under Chinese Han rule.

#### Distribution.

China (Taiwan) (Fig. 12).

### Ptomaphagus (s. str.) tingtingae sp. n.

Taxon classificationAnimaliaColeopteraLeiodidae

http://zoobank.org/D518FD86-9AD9-458D-972C-121655AF9A0D

Figs 3C–E ; 9B, D, F, I, J ; 10C, D ; 11

#### Type locality.

Taiwan, Fushan [福山, ca. 24°46'N, 121°30'E].

#### Type material.

**Holotype**: ♂, CHINA, Taiwan, Fushan [福山, ca. 24°46'N, 121°30'E], mouse carcass bait, III.2007, Wen-Bo Huang leg. (5#) (NTUC). **Paratypes**: 6♂♂7♀♀, same data as holotype (1♂1♀ in CCBW, 1♂1♀ in CJRZ, 1♂1♀ in CMNE, 1♂1♀ in CMPR and 2♂♂3♀♀ in NTUC); 7♀♀, same data as holotype except: IV.2007, (1#) (NTUC); 2♂♂1♀, Taiwan: Tai Pei Co. / Noi Dong [?Neidong=内洞, ca. 24°49'N, 121°32'E] Logging Road, / 850 m, 19.ii.2004, / Flight intercept trap. / leg. Chun Lin Li (ZMHB); 2♀♀, Taiwan: Tai Chun Co., / An Ma Shan [鞍馬山, ca. 24°16'N, 121°00'E], 2 km, / 24.-26.vi.2003 / Flight intercept trap. / leg. Chun Lin Li (ZMHB).

#### Description.

*Male*. EBL: 4.0–4.3 mm (4.1 mm in holotype). Length of different body parts: HL : AL : PL : ELL = 0.62 : 1.22 : 1.00 : 2.27 mm; width: HW : EW : PW : ELW = 1.00 : 0.11 : 1.55 : 1.68 mm. Proportion of antennomeres from base to tip in μm (length × width): 180 × 72, 134 × 66, 111 × 74, 73 × 79, 73 × 92, 60 × 111, 92 × 127, 47 × 125, 87 × 136, 101 × 140, 176 × 132.

*Habitus* (Fig. 3C, E) elongated oval, regularly convex and sublustrous. Well pigmented: mostly dark brown, head darker; mouthparts, basal three antennomeres and apical half of ultimate antennomere, protarsi, and apex of meso- and metatarsi somewhat yellowish. Dorsum continuously clothed with fine, recumbent, yellowish pubescence. Insertions of pubescence on dorsal surfaces of pronotum, elytra and femora aligned along transverse striolations; interspace between two striolations glabrous.

*Head transverse*, HW/HL = 1.6. Clypeofrontal suture absent. Clypeus with anterior margin slightly rounded. Compound eyes well developed, EW/HW = 0.1. Antennae (Fig. 11A) slender, AL/HW = 1.2; antennomere III a little shorter than II; VI with length/width = 0.5; XI pear-shaped.

*Pronotum* (Fig. 11B) transverse, widest just before hind angles, PW/PL = 1.6. Sides gently arched, gradually narrowing from posterior to anterior; hind angles projected backwards and acute. Posterior margin widely protruding in the middle part, distinctly emarginate near hind angles.

*Elytra* oval, widest at about basal 1/3, ELL/EW = 1.4. Sides weakly arched, gradually narrowing from widest part to apices, which narrowly rounded (Fig. 11G). Sutural striae present. Metathoracic wings fully developed.

*Prolegs* robust, with basal three protarsomeres (Fig. 11C) strongly expanded: TW/BTW = 1.2. Protibiae (Fig. 11C) distinctly expanded towards apex. Profemora rather broad. Mesotibiae arcuate, mesotarsi simply linear. Metatibiae slender and straight.

*Abdominal ventrite VIII* (Fig. 11I) narrowly round at posterior edge, though an inconspicuous median notch at the median. Genital segment (Fig. 8J) with very slender spiculum gastrale, protruding about 3/8 of its length beyond anterior edge of epipleurite IX.

*Aedeagus* (Fig. 9B) long and rather slender, with median lobe gradually narrowing towards narrowly lanceolate apical part and terminated by round knob in dorsal view; opening of genital orifice situated on dorsal surface, deeply cut inwards on preapical left margin of median lobe. Ventral surface of the apex of the median lobe (Fig. 9J) inserted with a row of 6 ventrally-oriented setae on the left side and a row of 5 ventrally-oriented setae on the right side. Parameres narrow, reaching about apical 1/6 of median lobe, each with 1 apical and 2 preapical setae, the apical one very shorter (Fig. 9F). In lateral view (Fig. 9D), median lobe regularly bent ventrad, gradually tapering apically. Endophallus with stylus quite slender, a cheliform complex just below the base of stylus, and a circular complex in basal region.

*Female*. Similar to male in general appearance (Fig. 3D), including elytral apices (Fig. 11H), but distinguished by the following characteristics: protarsi (Fig. 11D) simply linear; protibiae (Fig. 11F) only slightly narrower; abdominal ventrite VIII (Fig. 10D) regularly rounded at posterior edge; genital segment as shown in Fig. 10C: spermatheca (Fig. 10C) curved in distal part and coiled in proximal part.

#### Diagnosis.

See under Ptomaphagus (s. str.) chenggongi sp. n. above.

#### Etymology.

The specific epithet is dedicated to Miss Ting-Ting Song, the first author’s former colleague (Institute of Zoology, Chinese Academy of Sciences, Beijing, China), who did important primary work on Chinese *Leiodes* Latreille, 1796 (Leiodidae: Leiodinae).

#### Distribution.

China (Taiwan) (Fig. 12).

## Supplementary Material

XML Treatment for Ptomaphagus

XML Treatment for Ptomaphagus s. str.

XML Treatment for Ptomaphagus (s. str.) kuntzeni

XML Treatment for Ptomaphagus (s. str.) yasutoshii

XML Treatment for Ptomaphagus (s. str.) chenggongi

XML Treatment for Ptomaphagus (s. str.) tingtingae
